# Pollution Characteristics and Human Health Risks of Elements in Road Dust in Changchun, China

**DOI:** 10.3390/ijerph15091843

**Published:** 2018-08-27

**Authors:** Na Li, Weizheng Han, Jie Tang, Jianmin Bian, Siyue Sun, Tiehong Song

**Affiliations:** 1 Key Laboratory of Groundwater Resources and Environment, Ministry of Education, Jilin University, Changchun 130021, China; tangjie0724@163.com (J.T.); bianjianmin@126.com (J.B.); 2 Key Laboratory of Songliao Aquatic Environment, Ministry of Education, Jilin Jianzhu University, Changchun 130118, China; susiyue0626@126.com (S.S.); tiehongsong@ufl.edu (T.S.); 3 Changchun Institute of Urban Planning & Designing, Changchun 130031, China; blackhwz@163.com

**Keywords:** road dust, trace elements, enrich factor, average daily dose, cancer risk

## Abstract

Road dust, which contains trace elements and certain organic matter that can be harmful to human health, plays an important role in atmospheric pollution. In this paper, concentrations of 16 elements in the road dust of Changchun, China were determined experimentally. A total of 100 samples were collected using plastic brushes and dustpans, and the elements were analyzed by an inductively coupled plasma optical emission spectrometer (ICP-OES). It was indicated that the elements could be divided into major and trace elements. The concentration of trace elements followed the trend: mercury (Hg) > manganese (Mn) > zinc (Zn) > lead (Pb) > chromium (Cr) > copper (Cu) > vanadium (V) > arsenic (As) > nickel (Ni) > cobalt (Co) > cadmium (Cd). Contamination-level-assessment calculated by the geo-accumulation index (I_geo_) showed that the pollution-level ranged from non-contaminated to extreme contamination, while the calculations of enrichment factor (EF) showed that EF values exhibited a decreasing trend: Cd > Hg > As > Pb > Cu > Co > Zn > Ni > Cr > V > Mn > Mg > Fe > Sr > Ba. In our study, ingestion was the greatest exposure pathway for humans to intake trace elements by calculating the average daily dose (ADD) from three routes (ingestion, inhalation, and dermal contact). According to the health risk assessment results, the non-carcinogenic risks that human beings suffered from these elements were insignificant. Additionally, the hazard quotient (HQ) values were approximately one-tenth in the case of children. Meanwhile, the total excess cancer risk (ECR) was also lower than the acceptable level (10^−6^–10^−4^) for both adults and children.

## 1. Introduction

In recent decades, the rapidly growing economy of China has resulted in a dramatic change in human activities such as industrialization and urbanization [1,2,3,4]. As a result, serious environmental problems have emerged, especially air pollution, which has become one of the most significant environmental concerns of both the public and scientists for the past few decades. Researchers have analyzed atmospheric pollution sources such as coal combustion, vehicle emission, soil, construction, as well as road dust [5,6,7]. It is necessary to study the physical chemical properties of each source to obtain a more precise result of source analysis, such as element characteristics, organic component, polycyclic aromatic hydrocarbons (PAHs), polybrominated diphenyl ethers (PBDEs), and persistent organic pollutants (POPs) [8,9,10,11].

Heavy metal pollutants can enter the atmosphere and natural water systems through re-suspension and runoff, respectively [12,13,14,15]. Studies regarding the metal components of road dust have been conducted worldwide [16,17,18,19,20]. According to previous studies, the concentrations of elements in road dust are significantly different depending on land use, population diversity, human activities, vehicle situations, number of cars, commercial areas, park locations, etc. Furthermore, trace elements, especially heavy metals, may harm human health since they can enter the body through ingestion, inhalation, and dermal contact [21,22,23,24], and can even reach the lungs and alveoli. Lead (Pb), arsenic (As), nickel (Ni), cadmium (Cd), and chromium (Cr), which are likely to cause cancer [25], have been frequently reported in high concentrations and may cause an elevated cancer risk in both adults and children.

Changchun city is the capital of Jilin Province located on the Northeast Plain of China. The city area covers over 3619.42 km^2^, and the population was roughly 3,280,898 in 2015, an increase from approximately 1,643,000 in 1980. The temperature is low in winter and the heating period can last from October to the following March. The dominant wind directions are SW and NE through the year. Industrial work makes up the most significant land uses in this city, followed by residential and commercial usage. Since the first motor factory was built in Changchun, the automobile industry has become the most developed economic activity in the area. In addition, metallurgy, medical, machines and electronic manufacturing play an active part in the economy of Changchun city. The number of motor vehicles reached 1 million in 2011 [26]. This technical change can cause severe atmospheric pollution due to intense human activities and long-term heating in winter. Many studies have reported the physical chemical characteristics of aerosols and source appointment. Studies have been reported characteristics of elements in road dust throughout the world, but no research has been published in the area of study. The article supports the references of source allocation in Northern China.

The main objectives of this paper were: (1) to obtain the metal element concentrations of road dust in Changchun city; (2) to calculate the geo-accumulation index; (3) to determine the enrichment factor; (4) to analyze the average exposure dose of elements; (5) to obtain the value of hazard quotient (HQ) and hazard index (HI) for non-carcinogenic health risk; (6) to calculate the cancer risks of Pb, Ni, As, Cr and Cd. The overall goals were to identify and evaluate the pollution levels of the different elements in a northeastern city in China and to establish a comprehensive method for geochemical data interpretation.

## 2. Materials and Methods

### 2.1. Sample Collection

**Figure 1:**
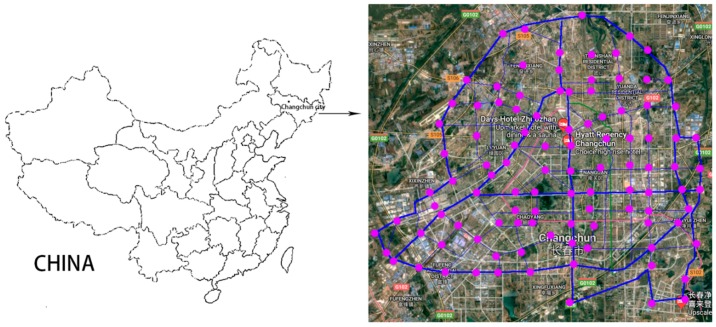
Sampling sites.

One hundred road dust samples were collected from Changchun in June 2017 (Figure 1). The sampling was not conducted on rainy or windy days, and the ground was dry, which was at least seven days after any rainfall. At each site, samples were collected from the indurative ground, with each sample weight being >100 g, by using plastic brushes and dustpans, which were stored in self-sealing polyethylene bags and brought to the lab as soon as possible. After being air-dried, the collected road dust was sieved with 100 μm nylon sieves to remove debris, hair, and leaves. The samples were measured by an inductively coupled plasma optical emission spectrometer (ICP-OES) and were stored at −20 °C before analysis. Sixteen typical elements were chosen to be tested. These were iron (Fe), manganese (Mn), Cr, Ni, Cd, magnesium (Mg), aluminum (Al), copper (Cu), zinc (Zn), Pb, barium (Ba), strontium (Sr), As, mercury (Hg), cobalt (Co), and vanadium (V). For the total concentrations, 0.1 g samples (three repeat parallel samples for each site) were measured on an electronic balance (Sartorius TE124S, Göttingen, Germany). Then, these were placed in Teflon tubes and digested using an 8 mL acid mixture ($V_{\mathrm{HCl}}:V_{{\mathrm{HNO}}_{3}}=1:3$). A microwave digestion apparatus (Topwave, Analytic Jena, Jena, Germany) was used to digest the samples, selecting the digestion program Soil EPA 3051 (175 °C, 10 min, 50 °C, 22 min). After cooling, the digested solutions were diverted to a volumetric flask and diluted to 50 mL. All the metals were determined by inductively coupled plasma optical emission spectrometer (ICP-OES, Prodigy XP, Leeman, Hudson, NH, USA). The standard curve was accepted when the correlation coefficients were close to 1. During the experimental process, ultra-pure water (electric resistivity: 18 MΩ × cm) was used for the dilutions. The used containers were dipped in liquid acid (10% HNO_3_ in volume) for 12 h, then rinsed to avoid potential cross-contamination of the samples.

### 2.2. Quality Assurance (QA) and Quality Control (QC)

Quality assurance (QA) and quality control (QC) were evaluated using duplicates, method blanks, and state first-level conventional materials (GBW GSS-5). Each sample was tested three times, and the results were accepted if the repeat sample analysis error was below 5%. The environmental procedures followed were by the Chinese Technical Specification for Soil Environmental Monitoring HJ/T 166-2004.

### 2.3. Contaminant Level Assessment

#### 2.3.1. Geo-Accumulation Index

The geo-accumulation index (I_geo_) assessment has been widely used to study metal enrichment by comparing each value of the trace elements to the crust concentration [27,28]. It was computed using the following equation:

(1) $$I_{\mathrm{geo}}=\log_{2}\frac{C_{x\left(\mathrm{sample}\right)}}{1.5\times C_{x\left(\mathrm{crust}\right)}}$$

C_x(sample)_ and C_x(crust)_ represent the concentrations of metal “x” for measuring and earth crust, respectively. Concerning the natural fluctuations and anthropogenic influence, the denominator must be multiplied by 1.5. In general, I_geo_ values > 1 may imply that the metal can be impacted by anthropogenic sources, which can be divided into seven classes as listed in Table 1.

#### 2.3.2. Enrichment Factor

The enrichment factor (EF) was selected to assess the enrichment and contamination levels of the elements [29]. EFs are calculated by the following formula:

(2) $${\mathrm{EF}=(C}_{n}{/C}_{\mathrm{Al}}{)}_{s}{/(C}_{n}{/C}_{\mathrm{Al}}{)}_{r}$$

(C_n_/C_Al_)_s_ is the ratio of the concentration of an element with that of Al at each sampling point, and (C_n_/C_Al_)_r_ is the same ratio of the background content in soils. The reference values can be selected as the Jilin Province background values. According to the EF values, five contamination levels were divided (Table 1).

#### 2.3.3. Exposure Dose

Exposure assessment reflects the considerations identified in the problem formulation, including exposure dose and hazard identification [30]. It can be divided into three routes (oral, respiratory, and dermal paths) for chemical penetration into the human body [31]. Consequently, the exposure dose assessment can also be divided into three parts: ingestion, inhalation and dermal contact [32,33], which can be calculated by the following equations:

(3) $${\mathrm{ADD}}_{\mathrm{ing}}=\frac{C\times \mathrm{CF}\times {\mathrm{IR}}_{S}\times \mathrm{FI}\times \mathrm{EF}\times \mathrm{ED}}{\mathrm{BW}\times \mathrm{AT}}$$

(4) $${\mathrm{ADD}}_{\mathrm{inh}}=\frac{C\times \frac{1}{\mathrm{PEF}}\times {\mathrm{IR}}_{a}\times \mathrm{ET}\times \mathrm{EF}\times \mathrm{ED}}{\mathrm{BW}\times \mathrm{AT}}=\frac{C\times {\mathrm{IR}}_{a}\times \mathrm{ET}\times \mathrm{EF}\times \mathrm{ED}}{\mathrm{PEF}\times \mathrm{BW}\times \mathrm{AT}}$$

(5) $$\begin{array}{ll} {\mathrm{ADD}}_{\mathrm{derm}} & =\frac{{\mathrm{DA}}_{\mathrm{event}}\times \mathrm{EF}\times \mathrm{ED}\times \mathrm{EV}\times \mathrm{SA}}{\mathrm{BW}\times \mathrm{AT}}\\ & =\frac{C\times \mathrm{CF}\times \mathrm{AF}\times \mathrm{ABS}\times \mathrm{EF}\times \mathrm{ED}\times \mathrm{EV}\times \mathrm{SA}}{\mathrm{BW}\times \mathrm{AT}} \end{array}$$

ADD_ing_, ADD_inh__,_ and ADD_derm_ represent the average daily exposure doses of metals via ingestion, inhalation, and dermal contact, respectively (mg/kg/day); C represents the concentration of metals in road dust (mg/kg); CF is the conversion factor (10^−6^ kg/mg); IR_s_ represents the ingestion rate of receptors (mg/day); FI represents the fraction ingested from a contaminated source (unitless); EF represents the exposure frequency (days/year); ED represents the exposure duration (years); BW represents body weight (kg); AT represents the averaging time (days), non-carcinogenic effects is ED × 365 days/year, and carcinogenic effects is 70 years × 365 days/year; IR_a_ represents the inhalation rate for receptor (m^3^/h); PEF represents the particulate emission factor (kg/m^3^); ET represents the exposure time (hours/day), DA_event_ represents the absorbed dose per event (mg/cm^2^/event), the calculation is C × CF × AF × ABS; AF represents the adherence factor from road dust to skin (mg/cm^2^/event); ABS represents the dermal absorption fraction; SA represents the skin surface area available for contact (cm^2^); and EV represents the event frequency (events/day). These parameters are shown in Table 2.

#### 2.3.4. Non-Carcinogenic Health Risk

Hazard quotient (HQ) and hazard index (HI) can be used to assess the non-carcinogenic health risk for both adults and children. HQ is the individual value for each element, and HI is the sum of HQ. These equations are as follows:

(6) $$\mathrm{HQ}=\frac{\mathrm{ADD}}{\mathrm{RfD}}$$

(7) HI=∑HQ

where ADD represents the ingestion, inhalation and dermal daily dose, which are mentioned above; RfD is a specific reference dose, which can be obtained from the Integrated Information Risk System (IRIS) [37]. If HQ > 1, it implies an adverse effect on human health. Otherwise, if HQ < 1, the results are the opposite. The safe limit of HI is also 1. If HI < 1, there is no significant risk of non-carcinogenic effects; otherwise it has the opposite effect.

#### 2.3.5. Risk Calculation

The reference values of carcinogenic risk through dermal exposure and ingestion were not provided by the USEPA, so it cannot calculate the excess cancer risks (ECR) on the two exposure pathways except for inhalation. Furthermore, the inhalation pathway can only consider the carcinogenic risk of metals. Aside from this point, not all metals can cause cancer. In our study, 16 elements were tested, but only Cr, Ni, As, Cd, Pb were related to cancer (IRIS) [37]. Hence, only five elements were considered when we calculated the cancer risk. The ECR for an exposed receptor can be assessed with the following equation [18]:

(8) $${\mathrm{ECR}}_{\mathrm{inh}}=\mathrm{SF}\times {\mathrm{ADD}}_{\mathrm{inh}}=\frac{\mathrm{IUR}\times \mathrm{BW}}{{\mathrm{IR}}_{a}}\times \frac{C\times {\mathrm{IR}}_{a}\times \mathrm{ET}\times \mathrm{EF}\times \mathrm{ED}}{\mathrm{PEF}\times \mathrm{BW}\times \mathrm{AT}}\;=\frac{C\times \mathrm{ET}\times \mathrm{EF}\times \mathrm{ED}\times \mathrm{IUR}}{\mathrm{PEF}\times \mathrm{AT}}$$

SF represents the cancer slope factor; and IUR represents the inhalation unit risk. Usually, the default approach recommended for determining predictive cancer risk is a linear extrapolation from the exposures observed in the animal or human occupational study. The slope of this line is commonly called the slope factor, and the units are defined as IUR [30]. The IUR values of Pb, Ni, As, Cd and Cr are 1.2 × 10^−5^, 2.4 × 10^−4^, 4.3 × 10^−3^, 1.8 × 10^−3^, and 0.012 (µg/m^3^)^−1^, respectively [34]; ET represents the exposure time, which in this study was 8 h/day. The other parameters were the same as those mentioned earlier. The safe limit of ECR is the range 10^−6^–10^−4^.

## 3. Results and Discussion

### 3.1. Road Dust Metals Concentration

Metal concentrations of the road dust and the summary statistics of the study area are listed in Table 3. The metals can be classified into two groups: major and trace elements. Metal concentrations of the trace elements followed the sequence: Hg > Mn > Zn > Pb > Cr > Cu > V > As > Ni > Co > Cd. Comparing the CNEMC values, all concentrations of the major elements were relatively lower; on the other hand, the concentrations of the trace elements were higher, except for Mn and V. Furthermore, the mean concentrations of Cd, Pb, As, and Hg were 214.3, 3.29, 5.97, and 14,000 times that of the CNEMC values, respectively; this was significantly higher than the other elements, which may be derived from anthropogenic sources.

The statistical characteristics included standard deviation (SD), the coefficient of variation (CV), skewness and kurtosis, which were calculated as presented in Table 3. CV is used to represent the discrete degree of data. CV ≤ 20%, low variability; 21% < CV ≤ 50%, moderate variability; 50% < CV ≤ 100%, high variability; CV > 100%, very high variability. In this study, concentrations of Ba, Cd, Pb, As, and Hg indicated high variability. The metals of Cd, Pb, As, and Hg varied widely between the different sampling sites, which further indicated anthropogenic sources. All these metals are related to traffic, state of the roads, tires, and brake wear, lubricants and paints and fuels [13,40,44]. The concentrations of Mn and Cr showed small differences while the others showed moderate variability.

The concentrations of the elements in road dust in this study were compared to different sites domestic and overseas, which are also presented in Table 4. Only the accumulation of Cr was lower than in other cities. The mean levels of Cd, As, and Hg were significantly higher than those in other sites.

### 3.2. Geo-Accumulation Index

**Figure 2:**
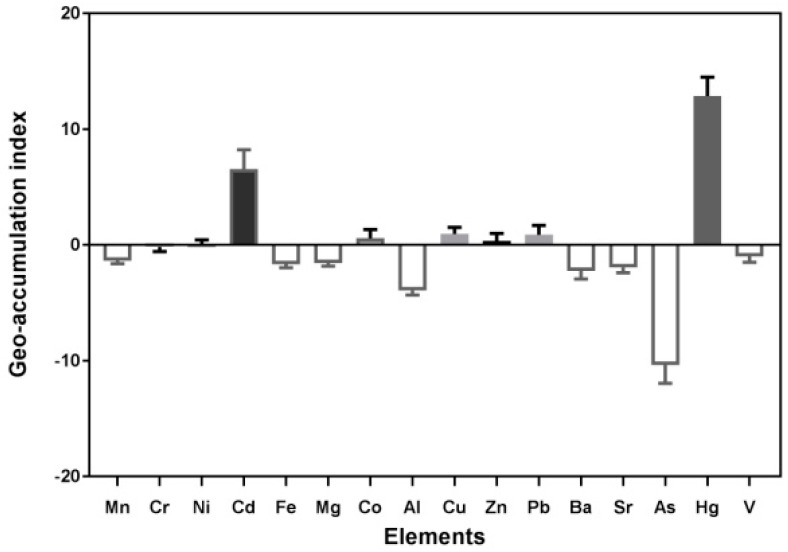
Boxplots of the geo-accumulation index (I_geo_) for 16 elements in road dust.

The I_geo_ values of elements were calculated in the road dust of Changchun, which are listed in Figure 2. The mean I_geo_ values of the elements followed the decreasing trend: Hg (12.87) > Cd (6.56) > Cu (0.96) > Pb (0.88) > Co (0.58) > Zn (0.36) > Ni (0.10) > Cr (−0.16) > V (−0.98) > Mn (−1.37) > Mg (−1.55) > Fe (−1.66) > Sr (−1.95) > Ba (−2.24) > Al (−3.94) > As (−10.37). The I_geo_ value of Hg was the highest, followed by Cd and Cu. According to the classifications and other studies [15,22,40], the average I_geo_ values indicated no contamination of the road dust by Mn, Cr, Fe, Mg, Al, Ba, Sr, As and V; no contamination to moderately contaminated by Ni, Co, Cu, Zn, and Pb; and extremely contaminated by Hg and Cd.

In addition, there was a wide range of values of elements presented in Figure 2, that indicated that the element contamination values varied by the diverse population, urban construction, traffic source, demolition activities, residential burning, industrial distribution, and other factors [43,45].

### 3.3. Enrichment Factor

**Figure 3:**
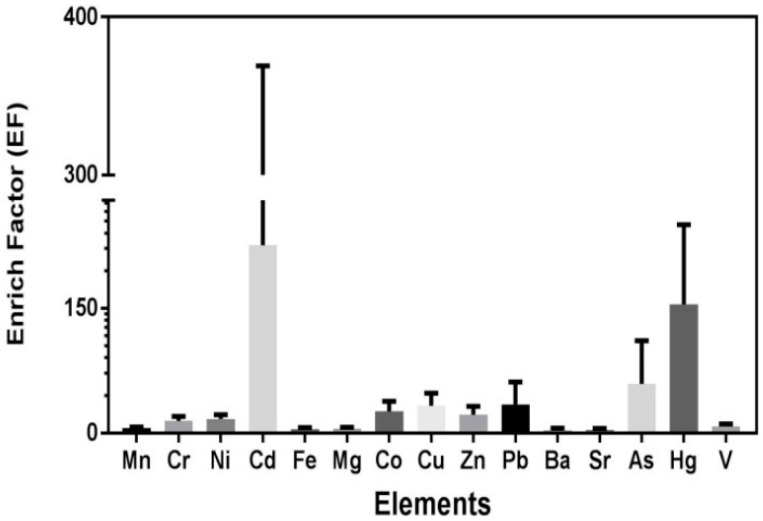
Boxplots of the enrichment factors (EF) for 16 elements in road dust.

The element of Al was selected as a reference value, and the results are presented in Figure 3. The EF of Cd, As, Hg was 18.31–384.22, 0.82–211.44, and 58.32–285.39, with the average of 225.55, 68.38, and 150.46, which indicated extremely high enrichment. The EF of Co, Cu, Zn, and Pb were 8.26–56.25, 9.15–94.22, 3.28–63.01 and 5.40–196.57, with an average of 26.20, 32.86, 22.01, and 34.53, respectively, which revealed a very high enrichment. The elements of Mn, Cr, Ni, Fe, and Mg showed significant enrichment, with the mean value ranging from 5–20. Ba and Sr were considered to originate primarily from natural sources, while the mean EF values were 3.68 and 4.2, respectively. The mean EF values exhibited the increasing trend: Ba < Sr < Fe < Mg < Mn < V < Cr < Ni < Zn < Co < Cu < Pb < As < Hg < Cd. The trends revealed that the carcinogenic elements had a higher enrichment than others, which showed significant anthropogenic sources.

### 3.4. Exposure Dose

**Figure 4:**
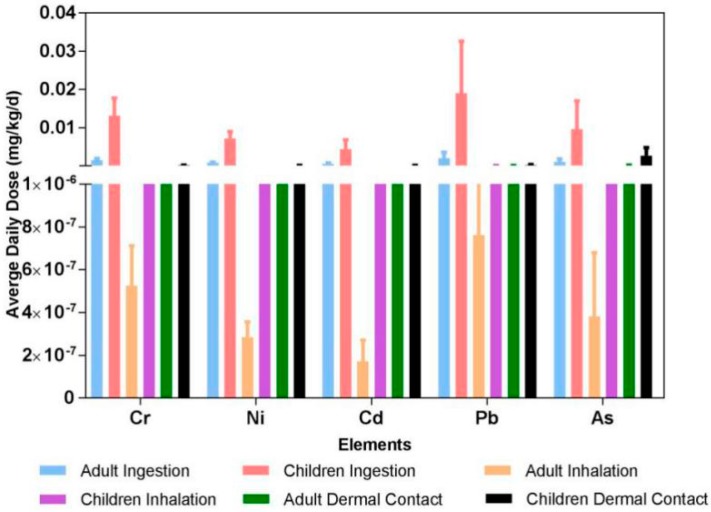
Average daily exposure dose for carcinogenic elements.

Table 5 shows the average daily dose (ADD) for the non-carcinogenic elements of road dust through three different exposure pathways, which revealed the same trend in adults and children, ADD_ing_ > ADD_derm_ > ADD_inh_. This situation was similar to what is seen in previous studies [22,23,44,46]. The ingestion exposure pathway is 2 to 4 orders higher than the other two types. Among the ADD of road dust, the elements Fe and Al had the maximum dose, while Cd had the minimum dose. According to the calculations, the total ADD for children was 5.2 times higher than adults, which indicates that children are exposed to more elements than adults. However, as there are various components in road dust, such as polycyclic aromatic hydrocarbons (PAHs), polybrominated diphenyl ethers (PBDEs), persistent organic pollutants (POPs), a higher dose does not necessarily indicate a higher risk while ignoring the assessment of others. Only five elements (Cr, Ni, Cd, Pb and As) had different carcinogenic effects on humans through IRIS, so the ADD for the carcinogenicity of the above five elements could be calculated, which are shown in Figure 4. It had the same trend in children and adults when compared to the non-carcinogenic assessment.

### 3.5. Assessment of Carcinogenic Health Risk

The results for HQ and HI are listed in Table 6. Both adults and children followed a similar trend for HQ for all elements: HQ_ing_ > HQ_derm_ > HQ_inh_ except for Mn which followed HQ_inh_ > HQ_derm_ > HQ_ing__,_ and Co which followed HQ_inh_ > HQ_ing_ > HQ_derm_. The HQ_ing_ values of Co (11.7), Hg (7.93), Pb (2.13), As (219), and the HQ_derm_ values of As (1.7), Hg (94), and HQ_inh_ value of Mg (1.33) were beyond 1 in the cases of children. The HQ values for all elements were all below 1 except Hg in the case of adults related to the ingestion pathway. It was found that the HI was approximately 10 times higher in children than adults; therefore, the elements of Co, Hg, Pb, and As in road dust had adverse health effects in the children group, while it existed in the Hg for adult ingestion.

### 3.6. Assessment of Cancer Risk

**Figure 5:**
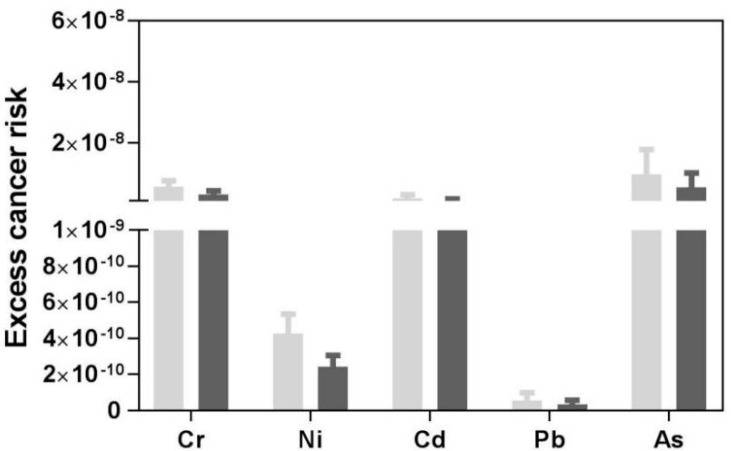
Excess Cancer Risk (ECR) of carcinogenic elements in road dust.

ECRs for carcinogenic risk via the inhalation pathway were calculated, and the results are shown in Figure 5. According to the IRIS, the ECRs for Cr, Ni, Cd, Pb and As were calculated in this study as were two additional forms of chromium persisting Cr (Ш) and Cr (VI). Only Cr (VI) has been established as carcinogenic. The value of Cr (VI) was used for calculating the carcinogenic health risk and was taken as one-seventh of the total mass of chromium in road dust since the ratio of Cr (VI) to Cr (III) is about 1:6 [34,47].

The trend of ECR was similar in both adults and children: As > Cr (VI) > Cd > Ni > Pb. The highest ECR for As related to adult inhalation was 2.24 × 10^−8^, which indicated that the value was under a permissible limit. Since the value was under the range limit of 10^−6^, contamination did not produce a serious carcinogenic risk.

## 4. Conclusions

The concentrations of 16 elements were divided into two groups: major and trace elements. Major elements are mainly from the Earth’s crust, and levels were lower than the background values, while the trace elements (except Mn and V) which could stem from anthropogenic sources were the opposite. The contamination level assessment was made by using the pollution indices, I_geo_, and EF. The results showed a wide range of values for different elements. Mn, Cr, Fe, Mg, Al, Ba, Sr, As, and V all exhibited non-contamination. Ni, Co, Cu, Zn, and Pb exhibited moderate contamination, and Hg exhibited extreme contamination. The EF results showed that the EF values exhibited a decreasing trend: Cd > Hg > As > Pb > Cu > Co > Zn > Ni > Cr > V > Mn > Mg > Fe > Sr > Ba. Cd, As, and Hg exhibited extremely high enrichment. Co, Cu, Zn, and Pb exhibited very high enrichment. Moreover, other elements exhibited significant enrichment.

The ingestion exposure was the highest for both adults and children, followed by dermal contact and inhalation. It showed the same decreasing trend for adults and children except for Mn and Co. The health risk analysis showed that Co, Hg, Pb, and As had the potential to cause non-carcinogenic risks in children. In contrast, the values for adults were well within the safe limit, which is approximately one-tenth that of children. According to the calculations of carcinogenic risk, the trend of ECR for carcinogenic factors was similar for both adults and children: As > Cr (VI) > Cd > Ni > Pb. The total ECRs were found to be under the adequate level for both adult and children. Although the results indicated no symbolic carcinogenic risk, people must pay more attention to the trace elements in road dust.

## Figures and Tables

**Table 1:** Contamination categories.

Geo-Accumulation Index			Contamination Categories Based on EF Values	
Class	I_geo_ value	Contamination level	EF values	Contamination categories
0	I_geo_ ≤ 0	Uncontaminated	EF < 2	Deficiency to minimal enrichment
1	0 < I_geo_ ≤ 1	Uncontaminated to moderately contaminated	2 < EF < 5	Moderate enrichment
2	1 < I_geo_ ≤ 2	Moderately contaminated	5 < EF < 20	Significant enrichment
3	2 < I_geo_ ≤ 3	Moderately to heavily contaminated	20 < EF < 40	Very high enrichment
4	3 < I_geo_ ≤ 4	Heavily contaminated	EF > 40	Extremely high enrichment
5	4 < I_geo_ ≤ 5	Heavily to extremely contaminated		
6	I_geo_ > 5	Extremely contaminated		

**Table 2:** The value of parameters for exposure dose.

Parm.	Unit	Class	Value	Parm.	Unit	Class	Value
IR_S_	mg/day	Adults	30 ^a^	IRa	m^3^/h	Adults	7.63
		Children	60 ^a^			children	20
EF	days/year	Adults	350 ^b^	ABS	unitless	Arsenic	0.03 ^b,c^
		Children	350 ^b^			others	0.001 ^d,c^
ED	years	Adults	30 ^b^	AF	unitless	Adults	0.07 ^b^
		Children	6 ^b^			children	0.2 ^b^
AT	days	Non-carcinogenic effects	ED × 365 ^b^	ET	h/day	Adults	14
		Carcinogenic effects	ED × 70 ^b^			children	8
BW	kg	Adults	70 ^b^	SA	cm^2^	Adults	5700 ^b^
		Children	15 ^b^			children	2800 ^b^
CF	kg/mg	——	10^−6^	PEF	kg/m^3^	——	1.36 × 10^9^
FI	unitless	——	1 ^b^	EV	events/day	——	1 ^b^

^a^ [33]; ^b^ [34]; ^c^ [35]; ^d^ [36].

**Table 3:** Metal concentrations and summary statistics (mg/kg).

Elements	*n* ^a^	Range (mg/kg)	Mean ± SD (mg/kg)	Median (mg/kg)	CV	Skewness	Kurtosis	CNEMC Value ^b^ (mg/kg)
Major elements								
Fe	100	5.47 × 10^3^–2.46 × 10^4^	1.33 × 10^4^ ± 2.87 × 10^3^	1.28 × 10^5^	0.22	0.98	2.29	2.74 × 10^4^
Mg	100	2.30 × 10^3^–7.80 × 10^3^	3.54 × 10^3^ ± 7.67 × 10^2^	3.43 × 10^3^	0.22	2.26	9.44	6.8 × 10^3^
Al	100	3.12 × 10^3^–2.19 × 10^4^	6.08 × 10^3^ ± 2.21 × 10^3^	6.08 × 10^3^	0.36	4.21	26.63	5.95 × 10^4^
Ba	100	68.77–780.97	193.97 ± 126.71	150.97	0.65	2.34	5.95	529.00
Sr	100	25.22–140.86	75.74 ± 21.28	77.69	0.28	0.03	0.22	187.00
Trace elements								
Mn	100	249.15–594.94	375.64 ± 64.63	367.49	0.17	1.01	1.75	636.00
Cr	100	30.36–208.69	65.53 ± 23.21	62.52	0.17	1.01	1.75	46.70
Ni	100	14.84–86.37	35.47 ± 9.03	35.47	0.25	2.02	11.00	21.40
Cd	100	1.98–36.73	21.43 ± 12.29	28.83	0.57	–0.92	–1.14	0.10
Co	100	9.56–41.27	29.96 ± 11.67	36.70	0.39	–0.92	–1.11	11.90
Cu	100	17.37–135.59	53.73 ± 21.66	50.36	0.40	1.21	2.29	17.10
Zn	100	34.56–419.86	169.79 ± 70.56	162.22	0.42	0.82	1.20	80.40
Pb	100	23.94–385.35	94.99 ± 67.99	73.31	0.72	2.26	5.86	28.80
As	95	0.56–144.27	47.72 ± 37.09	36.32	0.78	0.69	–0.68	8.00
Hg	73	0.53–1127.24	560.90 ± 294.68	543.43	0.53	0.04	–0.83	0.04
V	100	17.55–79.85	54.41 ± 15.77	60.05	0.29	–0.72	–0.89	68.00

^a^ The *n* means effective data number; ^b^ China National Environmental Monitoring Center (CNEMC) [38].

**Table 4:** Elements concentrations (mg kg^−1^) in road/street dust of different cities in China and other countries.

City	Year	Mn	Fe	Mg	Al	Ba	Sr	Cr	Ni	Cd	Co	Cu	Zn	Pb	As	Hg	V	References
Baoji, China	2010	804.20	——	——	——	——	——	123.00	42.00	——	16.00	113.00	612.00	383.00	18.00	——	31.40	[29]
Tehran, Iran	2012	——	——	——	——	——	——	33.50	34.80	10.70	——	225.30	873.20	257.40	——	——	——	[7]
Nanjing, China	2013	646.00	3.42 × 10^5^	——	——	——	——	126.00	——	——	11.00	123.00	394.00	103.00	13.00	——	——	[39]
Isfahan, Iran	2015	——	——	——	——	——	——	82.13	70.04	2.14	13.93	182.26	707.19	393.33	22.15	——	——	[10]
Villavicencio, Colombia	2016	——	——	——	——	——	——	25.63	22.27	——	——	213.40	289.73	467.63	——	——	——	[40]
Thessaloniki, Greece		529.10	2.51 × 10^5^	9.50 × 10^3^	2.92 × 10^4^	——	——	187.30	95.71	0.59	9.55	526.20	671.00	191.00	13.20	——	53.70	[14]
Changchun, China	2017	375.64	1.33 × 10^5^	3.54 × 10^3^	6.08 × 10^3^	193.97	75.74	65.53	35.47	21.43	29.96	53.73	169.79	94.99	47.72	54.41	54.41	This study
Hefei, China		240.50	8.69 × 10^3^	5.35 × 10^3^	8.39 × 10^3^	12.20	——	139.30	28.60	——	7.20	41.60	130.10	0.90	2.00	——	31.40	[41]
Xi’an, China		510.50	——	——	——	——	——	145.00	30.80	——	30.90	——	268.60	124.50	——	——	69.60	[42]
Beijing, China	2018	553.73	280.65	——	——	——	——	92.10	32.47	0.59	——	83.12	280.65	60.88	4.88	0.16	——	[43]

**Table 5:** Average daily exposure dose for non-carcinogenic elements through ingestion, inhalation and dermal contact.

Type	Data	Mn	Cr	Ni	Cd	Fe	Mg	Co	Al	Cu	Zn	Pb	Ba	Sr	As	Hg	V
Ingestion																	
Adults	Min	1.02 × 10^−3^	1.25 × 10^−4^	6.10 × 10^−5^	8.12 × 10^−6^	2.25 × 10^−2^	9.44 × 10^−3^	3.93 × 10^−5^	1.28 × 10^−2^	7.14 × 10^−5^	1.42 × 10^−4^	9.84 × 10^−5^	2.83 × 10^−4^	1.04 × 10^−4^	1.77 × 10^−5^	4.64 × 10^−3^	7.21 × 10^−5^
	Max	2.44 × 10^−3^	8.58 × 10^−4^	3.55 × 10^−4^	1.51 × 10^−4^	0.10	3.21 × 10^−2^	1.70 × 10^−4^	9.01 × 10^−2^	5.57 × 10^−4^	1.73 × 10^−3^	1.58 × 10^−3^	3.21 × 10^−3^	5.79 × 10^−4^	5.93 × 10^−4^	4.63 × 10^−3^	3.28 × 10^−4^
	Mean	1.54 × 10^−3^	2.69 × 10^−4^	1.46 × 10^−4^	8.81 × 10^−5^	5.46 × 10^−2^	1.46 × 10^−2^	1.23 × 10^−4^	2.50 × 10^−2^	2.21 × 10^−4^	6.98 × 10^−4^	3.90 × 10^−4^	7.97 × 10^−4^	3.11 × 10^−4^	1.86 × 10^−4^	1.07 × 10^−3^	2.24 × 10^−4^
Children	Min	5.34 × 10^−3^	6.51 × 10^−4^	3.18 × 10^−4^	4.24 × 10^−5^	0.12	4.92 × 10^−2^	2.05 × 10^−4^	6.68 × 10^−2^	3.72 × 10^−4^	7.41 × 10^−4^	5.13 × 10^−4^	1.47 × 10^−3^	5.41 × 10^−4^	1.23 × 10^−5^	1.42 × 10^−3^	3.76 × 10^−4^
	Max	1.27 × 10^−2^	4.47 × 10^−3^	1.85 × 10^−3^	7.87 × 10^−4^	0.53	0.17	8.84 × 10^−4^	0.47	2.91 × 10^−3^	9.00 × 10^−3^	8.26 × 10^−3^	1.67 × 10^−2^	3.02 × 10^−3^	3.09 × 10^−3^	2.42 × 10^−2^	1.71 × 10^−3^
	Mean	8.05 × 10^−3^	1.40 × 10^−3^	7.60 × 10^−4^	4.59 × 10^−4^	0.29	7.59 × 10^−2^	6.42 × 10^−4^	0.13	1.15 × 10^−3^	3.64 × 10^−3^	2.04 × 10^−3^	4.16 × 10^−3^	1.62 × 10^−3^	9.69 × 10^−4^	5.57 × 10^−3^	1.17 × 10^−3^
Inhalation																	
Adults	Min	3.83 × 10^−7^	4.67 × 10^−8^	2.28 × 10^−8^	3.04 × 10^−9^	8.41 × 10^−6^	3.53 × 10^−6^	1.47 × 10^−8^	4.79 × 10^−6^	2.67 × 10^−8^	5.31 × 10^−8^	3.68 × 10^−8^	1.06 × 10^−7^	3.88 × 10^−8^	1.62 × 10^−9^	7.34 × 10^−8^	2.70 × 10^−8^
	Max	9.14 × 10^−7^	3.21 × 10^−7^	1.33 × 10^−7^	5.65 × 10^−8^	3.79 × 10^−5^	1.20 × 10^−5^	6.34 × 10^−8^	3.37 × 10^−5^	2.08 × 10^−7^	6.45 × 10^−7^	5.92 × 10^−7^	1.20 × 10^−6^	2.17 × 10^−7^	2.22 × 10^−7^	1.73 × 10^−6^	1.23 × 10^−7^
	Mean	5.77 × 10^−7^	1.01 × 10^−7^	5.45 × 10^−8^	3.29 × 10^−8^	2.04 × 10^−5^	5.44 × 10^−6^	4.60 × 10^−8^	9.35 × 10^−6^	8.26 × 10^−8^	2.61 × 10^−7^	1.46 × 10^−7^	2.98 × 10^−7^	1.16 × 10^−7^	6.95 × 10^−8^	3.99 × 10^−7^	8.36 × 10^−8^
Children	Min	4.68 × 10^−6^	5.71 × 10^−7^	2.79 × 10^−7^	3.72 × 10^−8^	1.03 × 10^−4^	4.32 × 10^−5^	1.80 × 10^−7^	5.87 × 10^−5^	3.27 × 10^−7^	6.50 × 10^−7^	4.50 × 10^−7^	1.29 × 10^−6^	4.74 × 10^−7^	8.10 × 10^−8^	2.12 × 10^−6^	3.30 × 10^−7^
	Max	1.12 × 10^−5^	3.92 × 10^−6^	1.62 × 10^−6^	6.91 × 10^−7^	4.63 × 10^−4^	1.47 × 10^−4^	7.76 × 10^−7^	4.12 × 10^−4^	2.55 × 10^−6^	7.89 × 10^−6^	7.25 × 10^−6^	1.47 × 10^−5^	2.65 × 10^−6^	2.71 × 10^−6^	2.12 × 10^−5^	1.50 × 10^−6^
	Mean	7.06 × 10^−6^	1.23 × 10^−6^	6.67 × 10^−7^	4.03 × 10^−7^	2.50 × 10^−4^	6.66 × 10^−5^	5.63 × 10^−7^	1.14 × 10^−4^	1.01 × 10^−6^	3.19 × 10^−6^	1.79 × 10^−6^	3.65 × 10^−6^	1.42 × 10^−6^	8.50 × 10^−7^	4.88 × 10^−6^	1.02 × 10^−6^
Dermal contact																	
Adults	Min	1.36 × 10^−5^	1.66 × 10^−6^	8.11 × 10^−7^	1.08 × 10^−7^	2.99 × 10^−4^	1.26 × 10^−4^	5.23 × 10^−7^	1.70 × 10^−4^	9.49 × 10^−7^	1.89 × 10^−6^	1.31 × 10^−6^	3.76 × 10^−6^	1.38 × 10^−6^	1.06 × 10^−6^	6.17 × 10^−6^	9.59 × 10^−7^
	Max	3.25 × 10^−5^	1.14 × 10^−5^	4.72 × 10^−6^	2.01 × 10^−6^	1.35 × 10^−3^	4.26 × 10^−4^	2.26 × 10^−6^	1.20 × 10^−3^	7.41 × 10^−6^	2.29 × 10^−5^	2.11 × 10^−5^	4.27 × 10^−5^	7.70 × 10^−6^	2.37 × 10^−4^	6.16 × 10^−5^	4.36 × 10^−6^
	Mean	2.05 × 10^−5^	3.58 × 10^−6^	1.94 × 10^−6^	1.17 × 10^−6^	7.26 × 10^−4^	1.94 × 10^−4^	1.64 × 10^−6^	3.32 × 10^−4^	2.94 × 10^−6^	9.28 × 10^−6^	5.19 × 10^−6^	1.06 × 10^−5^	4.14 × 10^−6^	7.41 × 10^−5^	1.42 × 10^−5^	2.97 × 10^−6^
Children	Min	8.92 × 10^−5^	1.09 × 10^−5^	5.31 × 10^−6^	7.08 × 10^−7^	1.96 × 10^−3^	8.23 × 10^−4^	3.42 × 10^−6^	1.12 × 10^−3^	6.22 × 10^−6^	1.24 × 10^−5^	8.57 × 10^−6^	2.46 × 10^−5^	9.03 × 10^−6^	4.63 × 10^−5^	4.04 × 10^−4^	6.28 × 10^−6^
	Max	2.13 × 10^−4^	7.47 × 10^−5^	3.09 × 10^−5^	1.31 × 10^−5^	8.82 × 10^−3^	2.79 × 10^−3^	1.48 × 10^−5^	7.85 × 10^−3^	4.85 × 10^−5^	1.50 × 10^−4^	1.38 × 10^−4^	2.80 × 10^−4^	5.04 × 10^−5^	1.55 × 10^−3^	4.04 × 10^−4^	2.86 × 10^−5^
	Mean	1.34 × 10^−4^	2.35 × 10^−5^	1.27 × 10^−5^	7.67 × 10^−6^	4.76 × 10^−3^	1.27 × 10^−3^	1.07 × 10^−5^	2.18 × 10^−3^	1.92 × 10^−5^	6.08 × 10^−5^	3.40 × 10^−5^	6.94 × 10^−5^	2.71 × 10^−5^	4.85 × 10^−4^	9.30 × 10^−5^	1.95 × 10^−5^
Total exposure																	
Adults		1.56 × 10^−3^	2.73 × 10^−4^	1.48 × 10^−4^	8.93 × 10^−5^	5.53 × 10^−2^	1.48 × 10^−2^	1.25 × 10^−4^	2.53 × 10^−2^	2.24 × 10^−4^	7.08 × 10^−4^	3.95 × 10^−4^	8.08 × 10^−4^	3.15 × 10^−4^	2.60 × 10^−4^	1.08 × 10^−3^	2.27 × 10^−4^
Children		8.19 × 10^−3^	1.42 × 10^−3^	7.73 × 10^−4^	4.67 × 10^−4^	0.29	7.72 × 10^−2^	6.53 × 10^−4^	0.13	1.17 × 10^−3^	3.70 × 10^−3^	2.08 × 10^−3^	4.23 × 10^−3^	1.65 × 10^−3^	1.45 × 10^−3^	5.67 × 10^−3^	1.19 × 10^−3^

**Table 6:** Hazard Quotient and Hazard Index for adults and children.

Type	Data	Mn	Cr	Ni	Cd	Fe	Mg	Co	Cu	Zn	Pb	Ba	Sr	As	Hg	V
Adults	HQing	1.10 × 10^−2^	8.98 × 10^−2^	7.29 × 10^−3^	8.81 × 10^−2^	——	0.10	6.16 × 10^−3^	5.97 × 10^−3^	2.33 × 10^−3^	0.11	3.99 × 10^−3^	5.19 × 10^−4^	0.65	7.63	3.19 × 10^−2^
	HQinh	1.15 × 10^−2^	3.52 × 10^−3^	2.65 × 10^−6^	3.29 × 10^−5^	——	0.10	8.06 × 10^−3^	2.05 × 10^−6^	8.70 × 10^−7^	4.15 × 10^−5^	2.08 × 10^−3^	——	——	9.99 × 10^−3^	——
	HQderm	1.12 × 10^−2^	1.43 × 10^−2^	1.94 × 10^−3^	2.34 × 10^−2^	——	——	1.02 × 10^−4^	1.55 × 10^−3^	1.55 × 10^−4^	9.89 × 10^−3^	2.16 × 10^−3^	——	0.26	14.50	3.30 × 10^−4^
	HI	3.37 × 10^−2^	0.11	9.23 × 10^−3^	0.11	——	0.211	1.43 × 10^−2^	7.51 × 10^−3^	2.48 × 10^−3^	0.12	8.23 × 10^−3^	5.19 × 10^−4^	0.92	22.10	3.23 × 10^−2^
Children	HQing	1.80 × 10^−2^	0.45	4.11 × 10^−2^	0.87	——	8.21 × 10^−3^	11.70	0.18	1.21 × 10^−2^	2.13	1.45 × 10^−2^	2.89 × 10^−3^	219.00	7.93	——
	HQinh	0.14	4.31 × 10^−2^	3.24 × 10^−5^	4.03 × 10^−4^	——	1.33	9.86 × 10^−2^	2.51 × 10^−5^	1.06 × 10^−5^	5.07 × 10^−4^	2.55 × 10^−2^	——	——	0.12	——
	HQderm	7.31 × 10^−2^	9.38 × 10^−2^	1.27 × 10^−2^	0.15	——	——	6.70 × 10^−4^	1.01 × 10^−2^	1.01 × 10^−3^	6.48 × 10^−2^	1.42 × 10^−2^	——	1.71	94.90	2.16 × 10^−3^
	HI	0.23	0.59	5.38 × 10^−2^	1.02	——	1.34	11.8	0.19	1.32 × 10^−2^	2.19	5.42 × 10^−2^	2.89 × 10^−3^	221.00	103.00	2.16 × 10^−3^
RfD	RfDing	0.14	3.00 × 10^−3^	2.00 × 10^−2^	1.00 × 10^−3^	——	0.14	2.00 × 10^−2 c^	3.70 × 10^−2^ ^b^	0.30	3.50 × 10^−3^ ^b^	0.20	0.60	3.00 × 10^−4^	3.00 × 10^−4^ ^d^	7.00 × 10^−3^ ^c^
	RfDinh	5.00 × 10^−5^	2.86 × 10^−5^ ^a^	2.06 × 10^−2^ ^b^	1.00 × 10^−3^ ^b^	——	5.00 × 10^−5^	5.71 × 10^−6^ ^c^	4.02 × 10^−2^ ^b^	0.30 ^b^	3.52 × 10^−3^ ^b^	1.43 × 10^−4^ ^c^	——	——	8.57 × 10^−5^ ^d^	——
	RfDderm	1.84 × 10^−3^ ^c^	2.50 × 10^−4^ ^b^	1.00 × 10^−3^ ^b^	5.00 × 10^−5^ ^b^	——	——	1.60 × 10^−2^ ^c^	1.90 × 10^−3^ ^b^	6.00 × 10^−2^	5.25 × 10^−4^ ^b^	4.90 × 10^−3^ ^c^	——	3.00 × 10^−4^	2.1 × 10^−6^ ^d^	9.00 × 10^−3^

^a^ [22]; ^b^ [19]; ^c^ [11]; ^d^ The other values is selected from the IRIS [37].
